# A modified rehabilitation paradigm bilaterally increased rat extensor digitorum communis muscle size but did not improve forelimb function after stroke

**DOI:** 10.1371/journal.pone.0302008

**Published:** 2024-04-11

**Authors:** Sally Caine, Mariam Alaverdashvili, Frederick Colbourne, Gillian D. Muir, Phyllis G. Paterson

**Affiliations:** 1 College of Pharmacy and Nutrition, University of Saskatchewan, Saskatoon, Canada; 2 Neuroscience and Mental Health Institute, University of Alberta, Edmonton, Canada; 3 Department of Psychology, University of Alberta, Edmonton, Canada; 4 Western College of Veterinary Medicine, University of Saskatchewan, Saskatoon, Canada; Harvard Medical School, UNITED STATES

## Abstract

Malnutrition after stroke may lessen the beneficial effects of rehabilitation on motor recovery through influences on both brain and skeletal muscle. Enriched rehabilitation (ER), a combination of environmental enrichment and forelimb reaching practice, is used preclinically to study recovery of skilled reaching after stroke. However, the chronic food restriction typically used to motivate engagement in reaching practice is a barrier to using ER to investigate interactions between nutritional status and rehabilitation. Thus, our objectives were to determine if a modified ER program comprised of environmental enrichment and skilled reaching practice motivated by a short fast would enhance post-stroke forelimb motor recovery and preserve forelimb muscle size and metabolic fiber type, relative to a group exposed to stroke without ER. At one week after photothrombotic cortical stroke, male, Sprague-Dawley rats were assigned to modified ER or standard care for 2 weeks. Forelimb recovery was assessed in the Montoya staircase and cylinder task before stroke and on days 5–6, 22–23, and 33–34 after stroke. ER failed to improve forelimb function in either task (p > 0.05). Atrophy of extensor digitorum communis (EDC) and triceps brachii long head (TBL) muscles was not evident in the stroke-targeted forelimb on day 35, but the area occupied by hybrid fibers was increased in the EDC muscle (p = 0.038). ER bilaterally increased EDC (p = 0.046), but not TBL, muscle size; EDC muscle fiber type was unchanged by ER. While the modified ER did not promote forelimb motor recovery, it does appear to have utility for studying the role of skeletal muscle plasticity in post-stroke recovery.

## Introduction

Stroke is a leading cause of adult disability [1]. Although some spontaneous and rehabilitation-induced recovery can be achieved [2], more than half of stroke survivors have hand and arm abnormalities that can persist chronically. These include weakness or paralysis [3], sensory loss [4–6], reduced or abnormal muscle activation [7], abnormal muscle synergies [8, 9], spasticity and stiffness [10], and diminished reach and grasp control [11, 12]. In contrast, stroke in pre-clinical rodent models often results in robust recovery. The development of more translational pre-clinical modelling that better reflects clinical stroke, such as inclusion of clinically relevant comorbidities, is key to addressing this disparity [13].

Malnutrition is a common stroke comorbidity that is associated with worse functional outcomes in clinical studies [14–17], and its prevention in preclinical studies enhances post-stroke motor function [18, 19]. An important question that has received less study is how nutritional status and neurorehabilitation interact to promote optimal functional recovery.

A preclinical rehabilitation model that could be used to study the interaction between nutritional status and rehabilitation of forelimb skilled reaching after stroke is enriched rehabilitation (ER). This rehabilitation paradigm consists of daily skilled reaching practice combined with environmental enrichment that incorporates social housing with free access to novel toys and a running wheel [20]. ER arose from the long-standing use of environmental enrichment, which promotes recovery in many settings. Meta-analyses have demonstrated that environmental enrichment enhances recovery on most tests of motor function, relative to conventional housing, after experimental stroke in rats and mice [21, 22]. However, the addition of daily reaching practice to the environmental enrichment (i.e. generating the ER paradigm) yields additional benefit for mitigating deficits in fine motor dexterity such as skilled reaching [23, 24].

A barrier to selecting the ER paradigm to investigate whether malnutrition interferes with rehabilitation of post-stroke reaching is that rodents are typically chronically food-restricted to motivate them to practise reaching for a food reward [20, 25]. Unfortunately, this chronic food restriction would confound strictly controlled malnutrition protocols [18, 19] that would be employed for such an interaction study. Thus, we modified the ER to motivate reaching practice with a short fast. The first study objective was to determine if a modified ER paradigm comprised of environmental enrichment and skilled reaching practice motivated by a short fast would improve post-stroke recovery of reaching and symmetry in forelimb use during spontaneous exploration relative to a group exposed to stroke without ER.

Promoting recovery after stroke has largely centred on brain plasticity, while data are limited on plasticity of stroke-affected skeletal muscle as a target for motor function recovery [26]. In humans, skeletal muscle undergoes structural and metabolic changes after stroke that can exacerbate disability [27, 28]. In hemi-paretic stroke, there is loss of lean muscle mass and increased intramuscular fat in both the paretic and non-paretic limbs, with the former affected more severely [29–31]. Atrophy of more fast-twitch Type II fibers and hypertrophy of slow-twitch Type I fibers in the paretic limb are described [31, 32]. Although these changes have been studied most in the lower extremities, there is also evidence for reduced muscle thickness in the upper limbs [33]. A shift to a greater proportion of fast-twitch fibers in the muscle on the stroke-affected side is commonly reported [27, 32, 34], although other patterns have been observed [35]. Many factors are thought to explain discrepancies in post-stroke muscle changes reported, such as the specific skeletal muscles studied with their varying composition of fast- versus slow-twitch fibers, whether the changes are reported relative to the nonparetic side or to healthy control limbs, age, level of inactivity, the time that has elapsed since the stroke, and the presence of comorbidities such as malnutrition [31, 32, 34, 36]. The existence of the latter can exert a major negative impact on muscle [37, 38] that could aggravate stroke-induced sarcopenia and impede neurorehabilitation efforts.

In preclinical models, little attention has been paid to investigating whether stroke- and rehabilitation-induced alterations in skeletal muscle mimic those of clinical stroke and contribute to post-stroke functional recovery. Such studies vary with respect to many of the factors outlined above that could influence muscle alterations after stroke [39–43]. Other influential factors are likely to include the confounding influence of the surgical procedures used to induce stroke and associated loss of lean tissue mass as well as the extent to which comparisons are made to both surgical sham controls and the non-stroke targeted limb. McDonald *et al*. [43] reported plantaris muscle atrophy in the stroke-targeted hindlimb after photothrombotic hindlimb sensorimotor cortex stroke in the rat that was further characterized as atrophy of fast-twitch Type IIb fibers and hypertrophy of Type I, Ia, and IIx fibers; the proportion of fiber types was unchanged. A second study of photothrombotic stroke in the hindlimb sensorimotor cortex described hypertrophy of Type 1 fibres only, with no change in the proportion of fiber types [42]. Our goal was to investigate how our modified ER would influence post-stroke forelimb skeletal muscle structure relative to functional recovery after cortical stroke. The second study objective was therefore to investigate if this modified ER would preserve forelimb muscle size and metabolic fiber type after stroke, as compared to standard care.

## Materials and methods

### Experimental design

**Fig 1:**
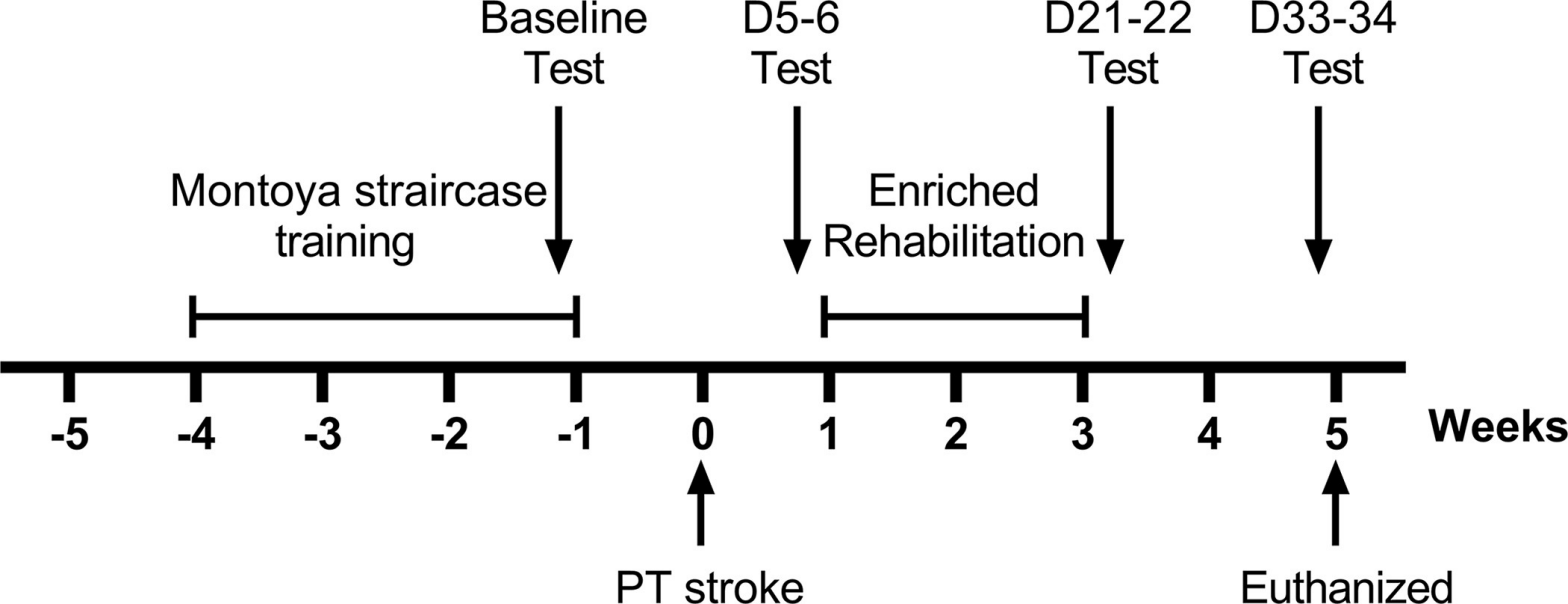
Timeline of experimental design. PT = photothrombotic; ER = Enriched Rehabilitation; SC = Standard Care. Test refers to the Montoya staircase and cylinder tasks.

Fig 1 shows the experimental design. Thirty-two male Sprague-Dawley rats (9 weeks old; Charles River Canada, QC, Canada) were randomly assigned to cage and treatment by using the random number generator in Excel. They were housed in groups of 4 in large standard polycarbonate cages (width 37.5 cm; length 48.0 cm; height 21.0 cm) in a temperature and humidity-controlled room with a 12-hour light/dark cycle. Rats were acclimatized to rat chow (Prolab RMH 3000 no. 0001495; LabDiet) for 5 days before assignment to a modified American Institute of Nutrition (AIN)-93M control diet (no. 100879; Dyets Inc.) [37]. Rats were handled for ~5 min daily in the behavioural testing facility for 5 days prior to 4 weeks of training in the Montoya staircase; rats were food-restricted to motivate the training, and then returned to ad libitum feeding. After an additional week, photothrombotic stroke was induced in all rats, which were by then 16 weeks of age. At day 7 after stroke, rats received either enriched rehabilitation (ER, n = 16) or standard care (SC, n = 16) treatment for 2 weeks. ER consisted of reaching practice in a modified Montoya staircase and overnight housing in an enriched environment (5 days/week) [44, 45]. For the primary experimental endpoint, rats were tested for forelimb reaching ability in the Montoya staircase test over 2 days at baseline (8–9 days prior to stroke), after stroke (day 5–6), and both immediately (day 21–22) and 2 weeks after (day 33–34) the end of ER. The cylinder test was performed on the second day of each test period. Brain was collected on day 35 for assessment of infarct volume; forelimb extensor digitorum communis (EDC) and triceps brachii long head (TBL) muscles were obtained to measure cross-sectional area and metabolic fiber composition. Food intake was measured daily and body weight every 1–2 days throughout the experiment. All experimental procedures were approved by the University of Saskatchewan’s Animal Research Ethics Board (Animal Use Protocol #20100148), were performed in accordance with the Canadian Council on Animal Care guidelines for humane animal use, and followed the ARRIVE guidelines [46].

### Photothrombotic stroke

Photothrombotic stroke was induced during the light phase as previously described [47]. The infarct was produced in the motor cortex contralateral to the forelimb that retrieved the highest average number of pellets over the last 8 trials of Montoya staircase training (see below). Rats were anaesthetised with 5% isoflurane in O_2_, secured in a stereotactic frame, and maintained on 1.75–3.00% isoflurane in 40% O_2_/60% N_2_O. Core temperature was maintained throughout the surgery by using a rectal probe with a homeothermic warming pad set to 37°C (RightTemp, Kent Scientific). Heart rate, respiration and oxygen saturation were monitored using a MouseOx Plus Pulse Oximeter and Physiological monitor (STARRLife Sciences Corp). The skin above the skull was incised, and a high-speed bone drill was used to thin a 4 mm × 4 mm square region of the skull over the caudal area of the motor cortex that controls forelimb function; the region was from +3 to -1 mm relative to bregma and 1–5 mm lateral to midline. Thinning was performed under a microscope until the vasculature was observed. A 4 mm × 4 mm aluminium mask was placed over the thinned area to restrict the illumination of the laser to the intended region. Rose Bengal (10 mg/kg) was injected via the tail vein followed by saline flushing. The thinned area was immediately illuminated for 10 minutes with a 532 nm Diode Pumped Solid State (DPSS) laser beam (~290 mW/cm^2^). The incision was sutured, and 2 mg/kg bupivacaine hydrochloride (Marcaine™, Hospira, Inc.) was injected subcutaneously at the incision site. Sterile saline was given subcutaneously (10 ml/kg/hr) to prevent dehydration. Rats were monitored closely following surgery for any seizures or signs of pain or discomfort; there were no complications.

### Behavioural training and testing

All behavioural training and testing occurred during the light phase.

#### Montoya staircase training and testing

Forelimb reaching and grasping ability was assessed with the Montoya staircase test [48]. For pre-stroke training, 3 sugar pellets (BioServ, Dustless precision pellets, 45 mg, banana flavor) per well were placed on each of the 7 steps on both sides of the apparatus. Rats underwent two 15-minute training sessions daily (5 days/week) for 4 weeks in the Montoya staircase. Rats were food restricted to approximately 80% of their ad libitum food consumption for the first 2 weeks of training and 85–90% for the remaining 2 weeks.

Pre-stroke baseline performance was calculated as the average of the last 4 training trials conducted over 2 days. Rats had to achieve a pre-stroke baseline performance of ≥ 10 pellets with either forelimb to be included in the post-stroke Montoya staircase analysis.

Testing in the staircase after the stroke was conducted after a 4 hour fast. Reaching performance was calculated as the average number of pellets successfully obtained over 4 trials on each of post-stroke days 5–6, 21–22, and 33–34; the 2 test sessions on each of these days were separated by 4 hours.

#### Cylinder test

Asymmetry in forelimb use was assessed during spontaneous exploration in the cylinder (20 cm diameter; 41 cm height) [49]. Rats were filmed for a minimum of both 5 minutes and 20 independent contacts with the cylinder wall; animals had to make at least 20 independent contacts with the cylinder wall in each testing session in order to be included in this analysis. Scoring of the video files was performed frame by frame by an experimenter blinded to the experimental conditions. The number of stroke-targeted, non-targeted, and bilateral forelimb contacts with the cylinder wall were counted, and percentage use of the stroke-targeted forelimb (contralateral to infarct) was calculated as [50]: $\left[\frac{forelimb\;contralateral\;to\;infarct+\frac{1}{2}\;bilateral\;contacts}{total\;contacts}\right]x\;100\%$

### Enriched rehabilitation (ER) versus standard care (SC)

ER consisted of two components [44, 45]. The first was reaching practice with the stroke-targeted forelimb 4 times per day (15 minutes per session) in a Montoya staircase modified to allow unlimited access to reach for sugar pellets. A fast of approximately 4 hours was used to encourage the rats to reach during the practice sessions. The second was housing of 4 rats/cage in an enrichment cage (61cm × 61.5 cm × 36 cm) with access to a running wheel, hammock, tube, ramp, and novel toys for 12 hours during the dark phase for 5 days/week; the same 4 rats were returned to a standard cage (59.5 cm × 38 cm × 20 cm) during the light phase. The toys were changed twice weekly, and the location of the food and water was varied daily. Running wheels in the enrichment cages were fitted with a bicycle wheel counter (Cateye Velo 7 CC-VL520); the distance run was recorded on a cage basis per night and corrected to account for the smaller circumference of the running wheel. SC consisted of housing 4 rats/cage (59.5 cm × 38 cm × 20 cm) with a single PVC tube. SC rats were fasted for the same length of time as the ER rats and were similarly moved to the behavioural testing suite but did not undergo any reaching practice. They received access to the same number of sugar pellets as retrieved by the ER rats during each reaching practice session.

### Infarct volume

Under isoflurane anesthesia, the rat was decapitated, and the head was directly plunged into liquid nitrogen before storing at -80°C. Subsequently, excess tissue was removed on dry ice with a dremel saw and the brain chiseled out of the skull in a cryostat as previously described [51]. This method was chosen to ensure the brains could also be used in a separate biochemical imaging study requiring unfixed, non-perfused tissue. The brain was sectioned coronally on a cryostat through the infarct at 14 μm without embedding in optimal cutting temperature compound (OCT). Serial sections 216 μm apart were stained with cresyl violet. Images were acquired on a digital slide scanner (Aperio Scan Scope SC, Leica Biosystems, USA) using a 20× objective, extracted at 30% the virtual size, and saved as JPEG files using Aperio Image Scope software (V11.2.0.780). The infarct area was measured by a blinded experimenter in ImageJ (National Institutes of Health, Bethesda MD–USA), and the infarct volume was calculated as: Totalinfarctvolume=averageareaofdamage×intervalbetweensections×numberofsections.

### Muscle preparation and analysis

#### Muscle dissection

Following decapitation, saline (0.9% NaCl) was perfused through the heart to remove blood from the forelimbs. Both forelimbs were removed, and the EDC and TBL muscles were dissected quickly on ice. A ~2 mm thick section was cut perpendicular to the fibers in the mid-belly region of each muscle, and the cross-sectional area was measured from digital images using ImageJ software. To reduce the degree of freezing artifact, the frozen samples were thawed, re-frozen in liquid nitrogen-cooled isopentane, and stored at -80°C [52]. Each frozen muscle was adhered to the cryostat chuck with a small amount of OCT (Tissue-Tek). Serial 10 μm sections were taken at -20°C in cross-section from the cut face of the muscle closest to the mid-belly section and captured on ProbeOn Plus slides (Fisher Scientific) before storing at -80°C until staining.

#### Muscle myosin heavy chain expression

Fiber typing by immunofluorescence staining of myosin heavy chain (MHC) expression [53] was performed on the EDC muscle for 6 rats from each group that were the most closely matched for body weight on the final day of the experiment. Primary mouse monoclonal antibodies, BA-F8 (MHC I), SC-71 (MHC IIa), BF-F3 (MHC IIb) deposited by S. Schiaffino and 6HI (MHC IIx) deposited by C. Lucas, were purchased from the Developmental Studies Hybridoma Bank (DSHB), created by the National Institute of Child Health and Human Development of the National Institutes of Health and maintained at The University of Iowa, Department of Biology, Iowa City, IA 52242. A cocktail containing BA-F8 (1:50), SC-71(1:600) and BF-F3 (1:100), followed by the corresponding cocktail of immunoglobin specific secondary antibodies, Alexa Fluor 350 IgG_2b_ (1:500), Alexa Fluor IgG_1_ (1:500) and Alexa Fluor 555 IgM (1:500, Invitrogen) were applied to the muscle sections. Thus, all four MHC fiber types were identified: Type I (blue), Type IIa (green), Type IIb (red) and Type IIx (unstained) (S1 Fig). To confirm the identity of the unstained fibers as Type IIx, another cocktail containing SC-71(1:600) and 6HI (1:50) was applied to an adjacent section to identify, respectively, Type IIa fibers (green) and Type IIx fibers (red).

Images of the entire muscle section were acquired using an Olympus VS110 slide scanner with a 20× objective. Each image was blinded to the experimenter, and a subset of the total muscle fibers was selected for analysis by randomly placing a grid of squares each measuring 100 μm × 100 μm spaced 300 μm apart over the muscle section (ImageJ). Muscle fibers that fell directly within the squares or crossed the top or right border of the square were measured while those that touched the left or bottom borders were excluded [54]. The number of pure and hybrid fibers were counted and expressed as a percentage of the total fibers counted. To assess the muscle fiber size, a mask of the muscle fibers was produced in Photoshop CS6 (Adobe Systems, Inc., San Jose. CA), and the cross-sectional area per fiber type was calculated using ImageJ.

### Animal exclusions

One rat was excluded from the SC group for all endpoints due to the absence of a visible infarct on cresyl violet stained sections; this was caused by a failed rose Bengal injection. Accordingly, analysis of body weight and physiological parameters measured during the surgeries had a sample size of n = 15 (SC) and n = 16 (ER).

Infarct volume was measured on 8 brains from the SC group and 9 brains from the ER group. This included all brains from animals that met behavioural training criteria minus 6 brains that were excluded due to sectioning difficulties. The latter loss occurred due to accidental fracture of the brain through the infarct region during snap-freezing or the chiseling step required to extract the frozen brains from the skulls.

For behavioural analysis, all rats with a visible infarct were included regardless of whether infarct volume could be measured. Animals that did not meet training criteria for the Montoya staircase were excluded from this analysis, leaving a sample size of n = 10 (SC) and n = 13 (ER). For cylinder analysis, excluding those animals that did not make 20 independent contacts with the cylinder wall during any test session allowed for analysis of n = 14 per group after exclusions. All excluded animals underwent all experimental procedures and remained as cage mates until the end of the experiment.

### Statistical analysis

**Fig 2:**
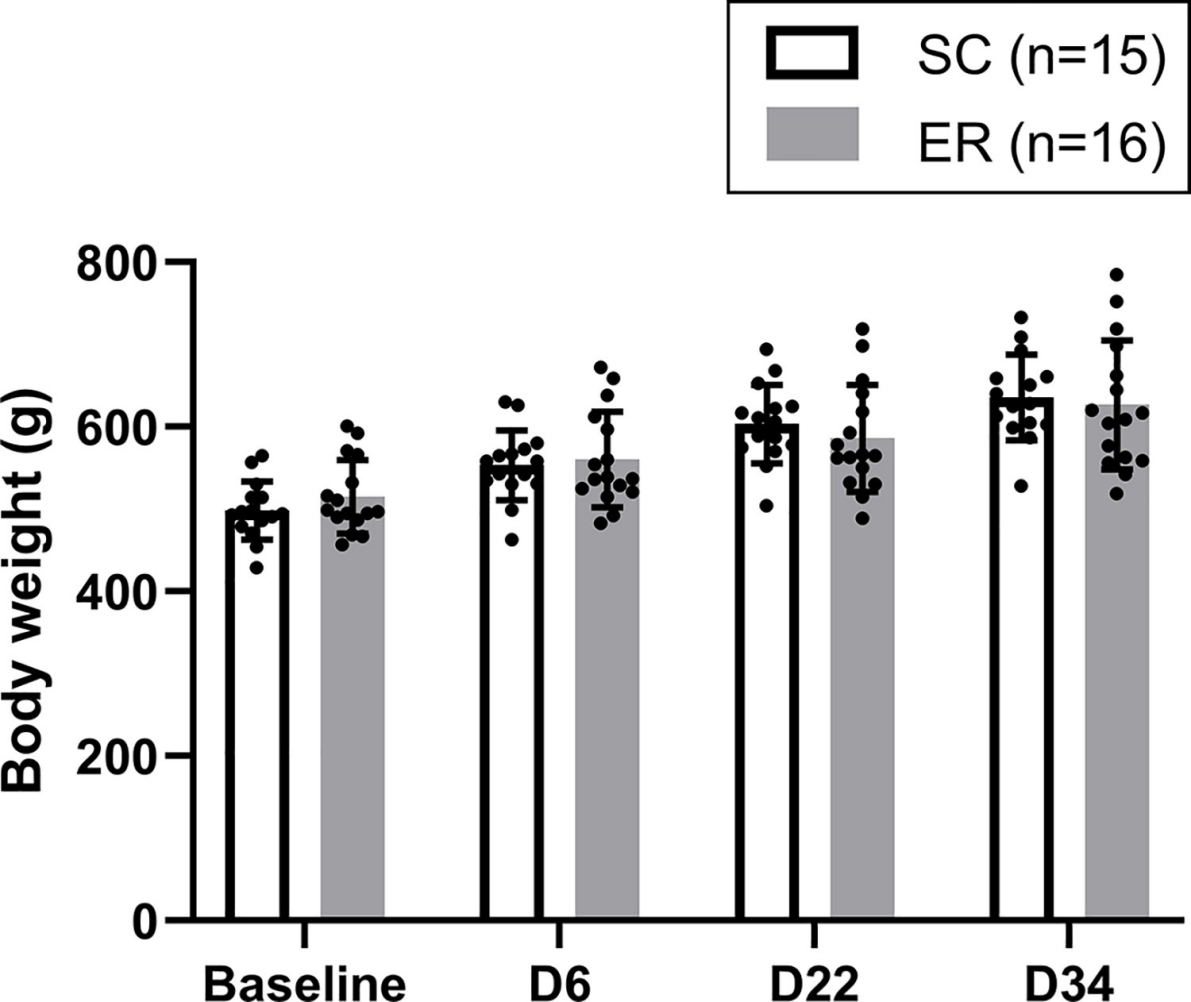
Pattern for temporal change in body weight (g) shown for behavioural testing days. Body weight significantly increased throughout the experiment. Although there was a significant interaction between treatment and time (P_interaction_ = 0.007, P_time_ <0.001, P_treatment_ = 0.980), post hoc independent t-tests did not reveal any differences between ER and SC groups at any time point (P>0.05). Data are shown as the mean ± SD with dots showing the individual data points. ER = Enriched Rehabilitation; SC = Standard Care.

**Fig 3:**
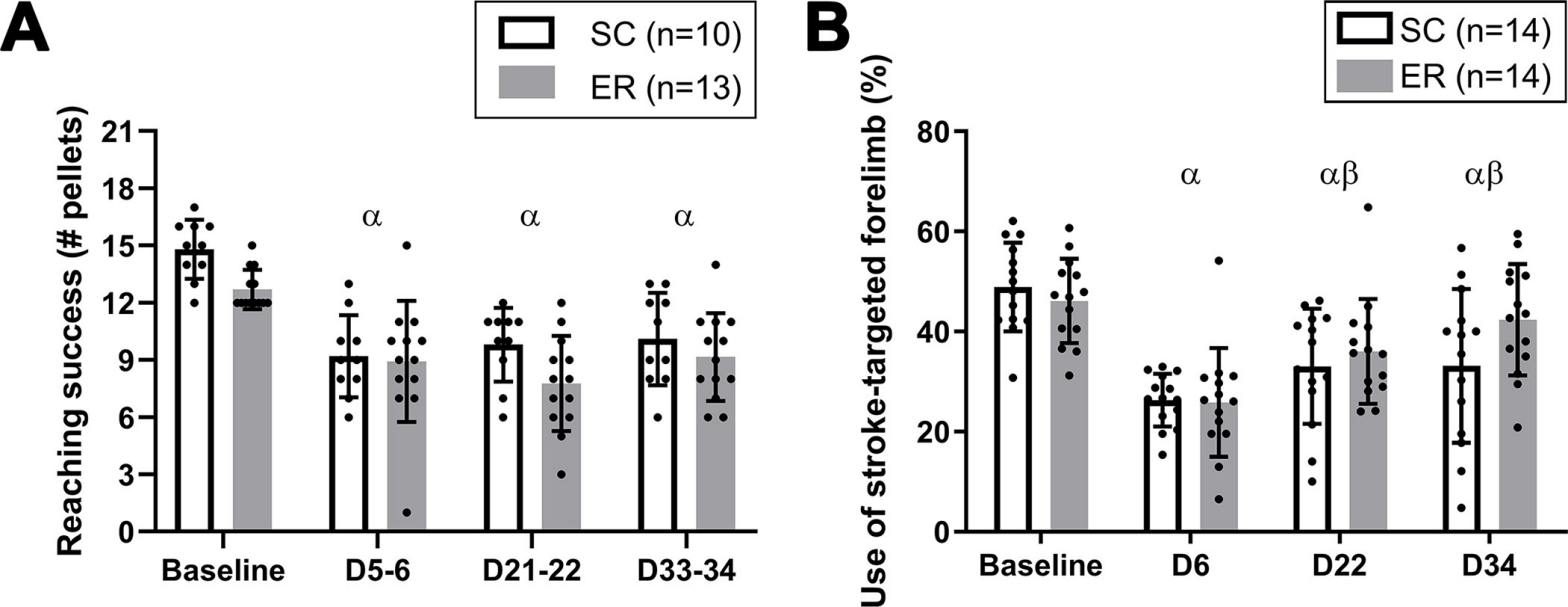
Enriched rehabilitation did not promote post-stroke recovery of reaching or forelimb use symmetry. (A) Reaching success with the stroke-targeted forelimb expressed as the number of pellets successfully reached in the Montoya staircase. ANOVA showed a significant effect of time with no interaction or effect of treatment: P_interaction_ = 0.236, P_time_ <0.001, P_treatment_ = 0.097. ^α^Indicates reaching success of the pooled groups is significantly lower than that at baseline (P<0.001). (B) Use of the stroke-targeted forelimb (%) during spontaneous exploration in the cylinder task. ANOVA analysis showed a significant effect of time with no interaction or effect of treatment: P_interaction_ = 0.123, P_time_ <0.001, P_treatment_ = 0.359. ^α^Indicates a significant decline in use of the stroke-targeted forelimb for the pooled groups as compared to baseline (P<0.05). ^β^Indicates a significant increase in use over that at D6 (P<0.05). Data are shown as the mean ± SD with dots showing the individual data points. ER = Enriched Rehabilitation; SC = Standard Care.

Statistical analyses were performed with SPSS Inc. (versions 25 and 26, Armonk, NY). Graphs were made in GraphPad Prism software for windows (version 8.2.1, San Diego, CA, USA) and composite images combined in Adobe Photoshop CC (Adobe Systems, Inc., San Jose. CA). Surgical physiological parameters and infarct measurements were analyzed by independent t-test or Welch’s t-test if Levene’s test for equality of variance was violated. Body weight and behavioural data were analysed using a repeated measures ANOVA, with Time as the within-subject variable and Treatment (SC or ER) as the between-subject factor. The Greenhouse-Geisser correction was used when the assumption of sphericity was violated. Follow up *post hoc* pairwise t-tests comparing each timepoint were performed with Bonferroni’s correction for multiple comparisons. The relationship between the quantity of sugar pellets consumed during the reaching practice in the ER group and the functional outcomes measured by the Montoya staircase and cylinder test was assessed with Pearson’s correlation coefficient. Muscle size and metabolic fiber type were analysed using a mixed ANOVA, with Forelimb (targeted or non-targeted) as the within-subject variable and Treatment (SC or ER) as the between-subject variable. A P-value of <0.05 was considered significant for all analyses, and noteworthy trends (P<0.10) are mentioned where appropriate. Data are expressed as the mean ± SD; Figs 2 and 3, and 5 also show individual data points.

## Results

### Physiological measurements during the surgical period

There were no significant differences in oxygen saturation, pulse or core temperature between the SC and ER groups during the surgical period (S1 Table).

### Body weight

Body weight significantly increased throughout the experiment, and there was a significant interaction between treatment and time (P_interaction_ = 0.007, P_time_ < 0.001, P_treatment_ = 0.980). However, post hoc independent t-tests did not reveal any differences between ER and SC groups at any time point (P > 0.05; Fig 2).

### Engagement in rehabilitation

Rats in the ER group ran on the running wheels a mean (± SD) total distance of 12.60 ± 9.94 km (range 0.65–2.73 km) per night on a cage basis (4 rats/cage). During the reaching practice component of ER, the total mean (± SD) amount of sugar pellets consumed from the four practice sessions per day was 5.0 ± 1.9 grams (~112 pellets).

### Infarct volume

There were no significant differences between the ER (n = 9) and SC (n = 8) groups for mean (± SD) infarct volume (SC—7.46 ± 1.51 mm^3^; ER—7.25 ± 1.37 mm^3^; P = 0.767), infarct location relative to bregma (SC—3.46 ± 0.74 to 0.54 ± 0.56 mm; ER—3.96 ± 0.27 mm to 1.07 ± 0.53 mm; P ≥ 0.065), or infarct placement lateral to midline (SC—0.70 ± 0.38 to 4.07 ± 0.50 mm; ER—0.53 ± 0.25 to 3.76 ± 0.30 mm; P ≥ 0.130).

### Behavioural outcomes

#### Montoya staircase

The number of pellets successfully reached in the Montoya staircase significantly changed with time, but there was no effect of treatment nor was there an interaction (P_interaction_ = 0.236, P_time_ < 0.001, P_treatment_ = 0.097; Fig 3A). Post hoc analysis showed a decline of ~30% in reaching success on day 5–6 in the pooled treatment groups after stroke as compared to baseline (P < 0.001). This remained significantly reduced on days 21–22 and 33–34, compared to baseline (P < 0.001), and reaching success did not significantly improve relative to day 5–6 (P > 0.05).

#### Cylinder test

There was a significant effect of time only on the use of the stroke-targeted forelimb in the cylinder task; treatment (ER versus SC) had no effect nor was there an interaction between time and treatment (P_interaction_ = 0.123, P_time_ < 0.001, P_treatment_ = 0.359; Fig 3B). Post hoc analysis on the pooled treatment groups showed a significant reduction in use of the stroke-targeted forelimb in the cylinder task on day 6 after stroke, relative to baseline (P < 0.0001). Use of the stroke-targeted limb increased on days 22 and 34 in both groups when compared with day 6 (P < 0.016) but remained significantly reduced relative to baseline (P < 0.01).

#### Correlations between reaching practice intensity and behavioural outcomes

**Fig 4:**
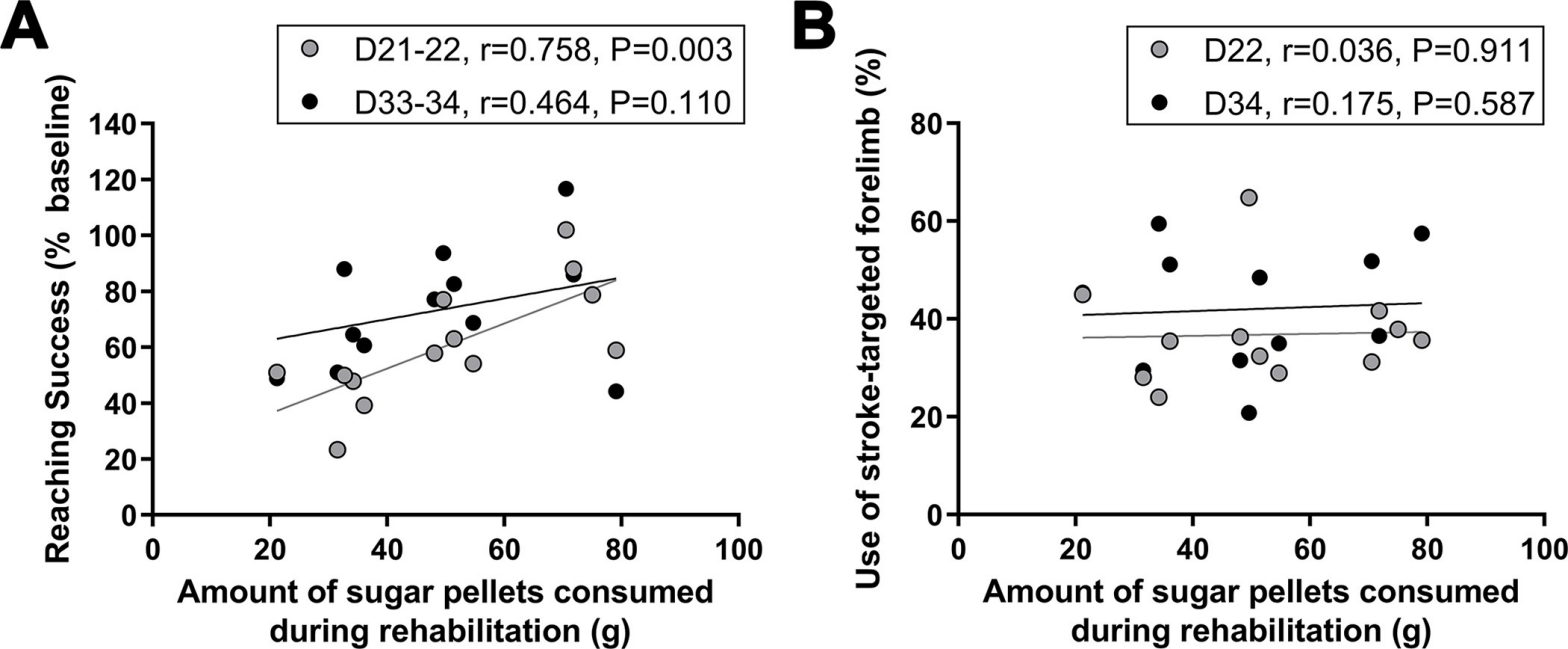
Correlations between reaching practice intensity and post-stroke reaching performance and forelimb use symmetry. The quantity of sugar pellets consumed during the reaching practice component of Enriched Rehabilitation correlated positively with post-stroke skilled reaching performance only immediately after ER ended (D21-22) but was not associated with forelimb use symmetry. Correlation of the amount of sugar pellets consumed (g) during the entire rehabilitation (ER) period with (A) reaching success in the Montoya staircase (n = 13) and (B) percent stroke-targeted forelimb use in the cylinder test (n = 12) across test days.

The quantity of sugar pellets consumed during the reaching practice component of ER correlated positively with reaching performance in the Montoya staircase on days 21–22 (P = 0.003) but not on days 33–34 (P = 0.110; Fig 4A). The amount of sugar pellets consumed during reach practice did not correlate with percent use of the stroke-targeted forelimb in the cylinder test at either time (P ≥ 0.587; Fig 4B).

### Muscle size and metabolic fiber type

**Fig 5:**
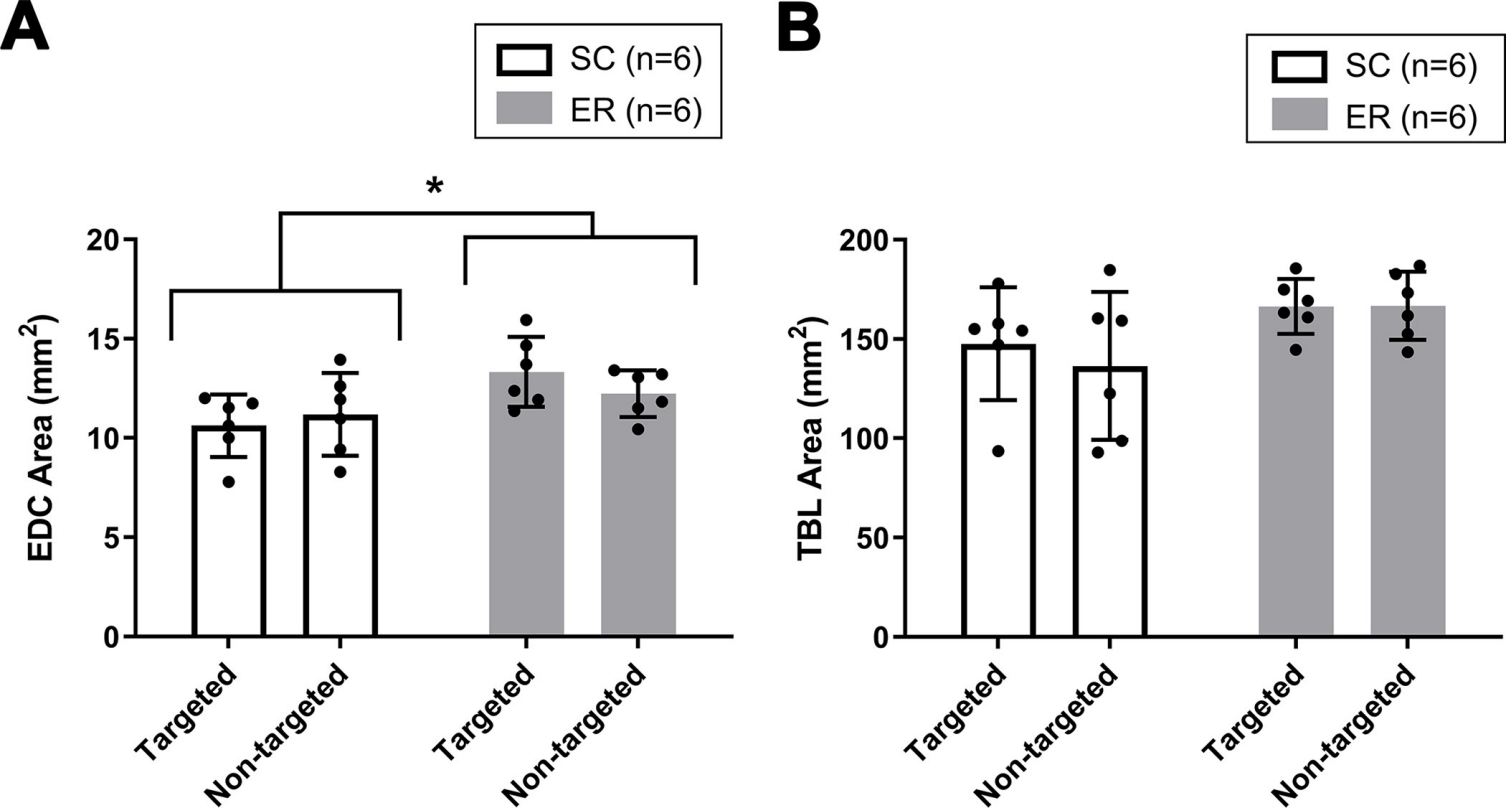
Effects of stroke and enriched rehabilitation on the size of selected forelimb muscles. Enriched rehabilitation increased cross-sectional area (mm^2^) at the mid-belly of the (A) extensor digitorum communis (EDC) muscle equally in stroke-targeted and non-targeted forelimbs (P_interaction_ = 0.133, P_forelimb =_ 0.627, P_treatment_ = 0.046). *Signifies the larger EDC muscle area for pooled forelimbs with ER treatment. Neither treatment nor forelimb affected the size of the (B) triceps brachii long head (TBL) muscle (P_interaction 0_ = 0.464, P_forelimb_ = 0.491, P_treatment_ = 0.086). Data are shown as the mean ± SD, with dots showing the individual data points. ER = Enriched Rehabilitation; SC = Standard Care.

Cross-sectional area of the mid-belly region of the EDC and TBL muscles is shown in Fig 5. ER increased EDC muscle size bilaterally, as compared to SC (P_interaction_ = 0.133, P_forelimb =_ 0.627, P_treatment_ = 0.046), but the TBL muscle was unaffected when analyzed by treatment and forelimb (P_interaction_ = 0.464, P_forelimb_ = 0.491, P_treatment_ = 0.086).

**Fig 6:**
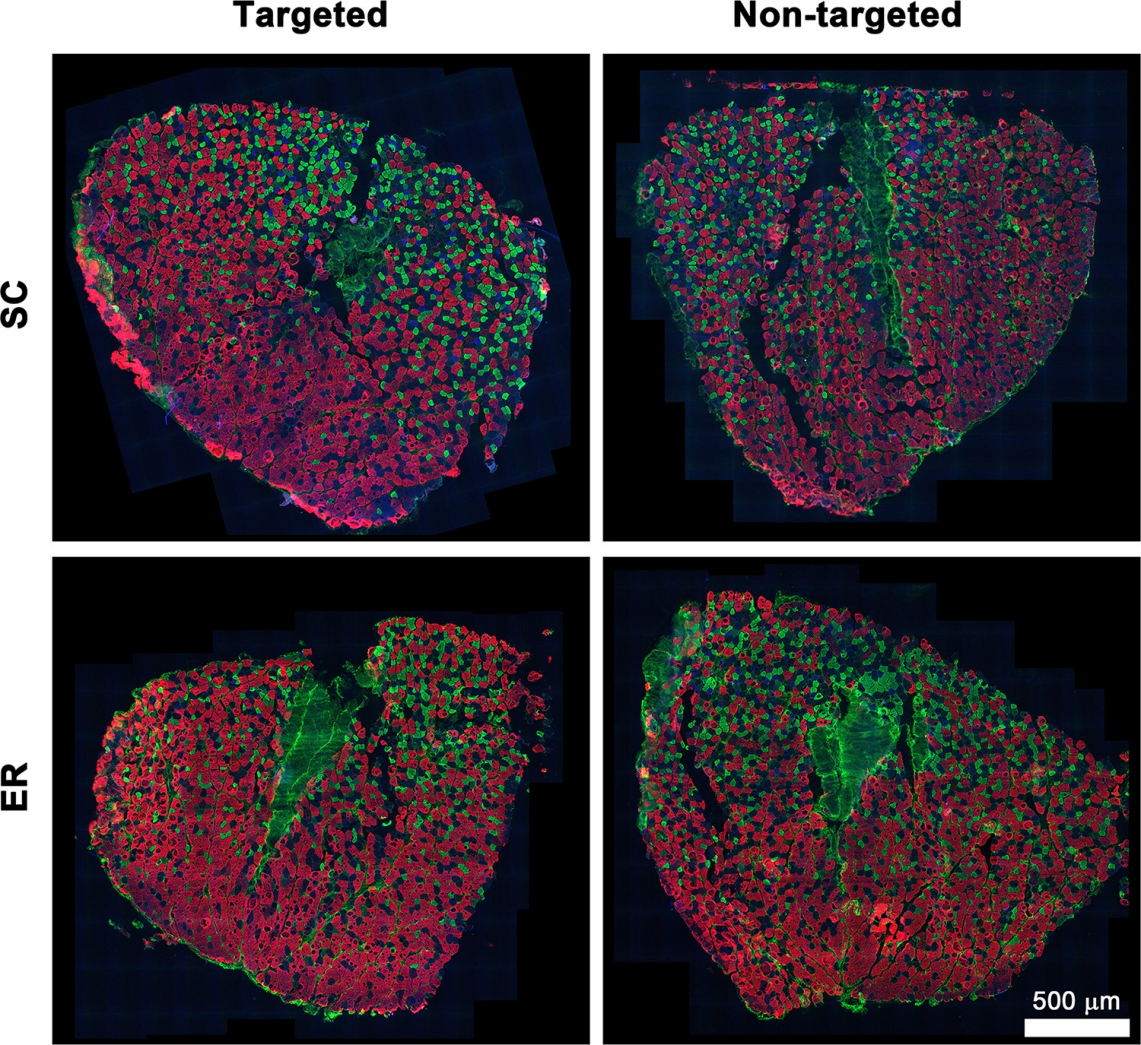
Distribution and relative abundance of muscle fiber types in extensor digitorum communis muscle. Representative immunofluorescence images of the myosin heavy chain distribution in muscle from stroke-targeted and non-targeted forelimbs from the Standard Care (SC) and Enriched Rehabilitation (ER) groups. Type I fibers (blue), Type IIa fibers (green), Type IIb fibers (red) and type IIx fibers (unstained/black).

Since there were no changes in the TBL muscle size, metabolic fiber type composition was assessed in the EDC muscle only for 6 animals per group that were weight matched. Representative immunofluorescence images showing the distribution and relative abundance of the EDC muscle fiber types are shown in Fig 6. Quantification of the relative abundance of muscle fiber types in the sampled regions, calculated number of muscle fiber types in the whole muscle section, muscle fiber size (area) in the sampled regions, calculated total area occupied by each fiber type in the whole muscle section, and area occupied by tendon in the muscle section are summarised in Table 1. Treatment had no effect on any endpoint, nor were there any significant interactions between treatment and forelimb (P > 0.05). However, there was one significant forelimb effect. The area occupied by hybrid fibers in the whole muscle section was significantly increased in the stroke-targeted forelimbs of the pooled treatment groups (P_interaction_ = 0.756, P_forelimb_ = 0.038, P_treatment_ = 0.896). Type IIa/x and Type IIb/x were the most abundant hybrid fibers (S2 Table). The Type IIb/x fibers occupied a significantly greater area in the whole muscle section in the stroke-targeted forelimbs of the pooled treatment groups (P_interaction_ = 0.962, P_forelimb_ = 0.039, P_treatment_ = 0.562). For the Type IIa/x hybrid fibers, there was no effect of treatment or forelimb, nor was there an interaction between treatment and forelimb for any of the measurements shown in S2 Table (P>0.05).

**Table 1:** Effects of stroke and enriched rehabilitation on extensor digitorum communis (EDC) muscle fiber types.

	Forelimb			
	SC Targeted	SC Non-targeted	ER Targeted	ER Non-targeted
	(n = 6)	(n = 6)	(n = 6)	(n = 6)
*Count (%)* ^*1*^				
Type I	1.70 ± 1.07	1.80 ± 1.77	0.88 ± 0.88	1.18 ± 0.78
Type IIa	17.97 ± 1.82	20.02 ± 5.26	17.47 ± 3.78	19.01 ± 4.31
Type IIx	21.35 ± 4.14	19.14 ± 3.01	23.32 ± 4.87	20.52 ± 5.23
Type IIb	47.16 ± 5.49	47.34 ± 5.16	48.18 ± 5.14	49.71 ± 8.50
Hybrids^*2*^	11.82 ± 1.78	11.64 ± 5.23	10.19 ± 1.35	9.58 ± 3.27
*Total counts* ^*3*^				
Type I	32 ± 27	21 ± 18	20 ± 23	22 ± 23
Type IIa	316 ±104	356 ± 244	337 ± 135	313 ± 209
Type IIx	377 ± 137	318 ± 209	461 ± 180	348 ± 222
Type IIb	820 ± 221	768 ± 418	917 ± 275	741 ± 240
Hybrids	212 ± 82	176 ± 108	195 ± 61	171 ± 141
*Fiber size (μm*^*2*^*)* ^*4*^				
Type I	1795 ± 1139	2267 ± 615	1845 ± 519	1949 ± 196
Type IIa	2361 ± 354	2263 ± 498	2596 ± 1200	2369 ± 690
Type IIx	3250 ± 523	3219 ± 678	3770 ± 1623	3526 ± 1068
Type IIb	5731 ± 605	5676 ± 1145	6696 ± 2222	6504 ± 1703
Hybrids	4192 ± 1146	3253 ± 797	3847 ± 2308	3707 ±1050
*Total area of fibers (mm*^*2*^*)* ^*5*^				
Type I	0.06 ± 0.06	0.05 ± 0.04	0.04 ± 0.05	0.04 ± 0.05
Type IIa	0.72 ± 0.16	0.74 ± 0.47	0.80 ± 0.28	0.68 ± 0.33
Type IIx	1.18 ± 0.33	0.93 ± 0.47	1.62 ± 0.64	1.12 ± 0.59
Type IIb	4.64 ± 1.13	4.08 ± 1.92	5.88 ± 1.62	4.57 ± 1.01
Hybrids	0.82 ± 0.27*	0.59 ± 0.28	0.77 ± 0.27*	0.59 ± 0.38
Tendon Area (mm^2^)	0.28 ± 0.16	0.77 ± 0.46	0.72 ± 0.41	0.74 ± 0.40

Data are shown as mean ± SD (n = 6/group). ER, Enriched Rehabilitation; SC, Standard Care. *Signifies that the area occupied by the hybrid fibers was significantly increased in the stroke-targeted forelimbs of the pooled treatment groups (P_interaction_ = 0.756, P_forelimb_ = 0.038, P_treatment_ = 0.896).

^1^The relative abundance of fiber types in the sampled regions.

^2^Includes Type I/x, IIa/x, IIb/x, I/IIb, I/IIa and IIa/b fiber types.

^3^The number of fiber types in the whole muscle (calculated from ^1^ and the muscle area) adjusted for tendon area.There were also some notable trends for stroke-targeted versus non-targeted forelimbs. The area occupied by both Type IIb fibers and Type IIx fibers in the whole muscle section showed a trend to be greater in the stroke-targeted forelimbs of the pooled treatment groups (Type IIb—P_interaction_ = 0.438, P_forelimb_ = 0.073, P_treatment_ = 0.248; Type IIx—P_interaction_ = 0.519, P_forelimb_ = 0.083, P_treatment_ = 0.202). Secondly, the stroke-targeted forelimb showed a trend towards an increased count % of IIb/x fibers of the pooled treatment groups, while ER showed a trend to decrease the count % of IIb/x fibers (P_interaction_ = 0.533, P_forelimb_ = 0.094, P_treatment_ = 0.051).

For the area occupied by tendon in the muscle section, there were no significant forelimb, treatment, or interaction effects (Table 1). However, ER showed a trend to protect against a reduction in tendon area in the stroke-targeted forelimb relative to the non-targeted forelimb, whereas SC did not (P_interaction_ = 0.075, P_forelimb_ = 0.052, P_treatment_ = 0.292).

## Discussion

We sought to establish whether a modified ER paradigm of skilled reaching practice motivated by a short fast coupled with the socialization and extensive activities of environmental enrichment would improve post-stroke forelimb motor recovery. However, no benefits of ER were observed, when compared to a group exposed to stroke but no rehabilitation, for either our primary endpoint, skilled reaching, or symmetry in spontaneous forelimb use.

The infarct size, extent of initial impairment, and rehabilitation intensity are critical predictors of post-stroke recovery of reaching ability in rats [55]. After stroke, and prior to rehabilitation, the animals initially showed a 30% decrease in reaching success in the Montoya staircase and 45% reduction in percentage use of the stroke-targeted forelimb for exploration in the cylinder test. Spontaneous recovery was evident only for the latter in either of the experimental groups over the 34-day test period. Damage to the caudal forelimb area and the adjacent region lateral to the rostral forelimb area of the motor cortex have been reported to best predict recovery of reaching in the Montoya staircase at 7 weeks after photothrombotic stroke, whereas damage in the area lateral to the caudal forelimb area predicted reduced recovery of spontaneous forelimb use in the cylinder test [56]. Unfortunately, the technical loss of some brain samples for measuring infarct volume from each of the ER and SC groups makes it difficult to assess the role of infarct size or placement in functional recovery in either experimental group. However, based on the 8 SC and 9 ER samples available, the rehabilitation that was initiated at one week following stroke did not influence infarct volume or placement.

The third critical predictor, rehabilitation intensity, may have significantly contributed to the failure of the modified ER to promote recovery of reaching. That is, an ~4 hour fast appears to have provided insufficient motivation for rats to engage adequately in reaching practice sessions. The average pellet retrieval of ~112 pellets per day fell below the estimated minimum rehabilitation intensity needed to promote recovery of reaching in the Montoya staircase [55, 57]. Our study design also did not assess the quality of the reaching movements during the practice sessions.

We also found no benefit of our modified enriched rehabilitation protocol for improving performance in the cylinder task. The latter measures more general motor function including proximal aspects of forelimb function, and even environmental enrichment without task-specific therapy often enhances recovery on such functions after stroke [21, 22]. Our negative finding may be related to the dose of cognitive, social, and physical stimulation in the enriched environment voluntarily chosen by the ER rats [23]. While the dose of environmental enrichment needed for stroke recovery is unknown, the synergistic effects of the components appear necessary for efficacy [24]. Our groups of four ER rats housed together voluntarily ran an average (± SD) of 12.60 ± 9.94 km per night during the rehabilitation period, but individual rat participation in this aerobic exercise was not measured. Similarly, each animal’s engagement with cage-mates, toys, and climbing opportunities is uncertain. Although there is no standardized treatment protocol for environmental enrichment [58], we aimed for more complexity by including a changing environment, multi-level cage, and running wheel. However, we did not provide continuous environmental enrichment, but rather exposure for twelve hours per day during the nocturnal period, and this may have contributed to the lack of efficacy. Although not the most likely explanation, the rats may have excessively increased reliance on the nontargeted limb in the environmental enrichment cage. Resulting plasticity in the non-damaged hemisphere may have promoted compensatory movement with the nontargeted forelimb, thus blocking some of the rehabilitation effect and inhibiting motor recovery of the stroke-targeted forelimb [59]. Most importantly, there are many uncertainties about the preclinical rehabilitation type, timing, intensity, and frequency required for efficacy with different infarct locations and severity [24, 58, 60, 61]. Failure to attain benefit is thus important to report for future meta-analyses needed to tailor preclinical rehabilitation protocols for these factors. Such analyses will be critical for achieving the ultimate goal of translating environmental enrichment to clinical practice.

For nutrition-related questions that necessitate avoidance of chronic food restriction to motivate reaching practice, it could be tested whether shifting the practice time to the rats’ dark and more active phase and providing longer periods of practice would allow utilization of such a fast to encourage reaching. Others have had to rely on the dark phase to ensure that rats reached above a critical rehabilitation threshold [57]. The fast is also likely to be more effective if introduced at the beginning of the dark phase, since rats consume the majority of food at the beginning and end of the active phase [62]. The future design of preclinical stroke studies with the goal of examining the interaction between nutritional status and rehabilitation in post-stroke recovery of motor skills could also utilize alternative rehabilitation paradigms that do not require food restriction to motivate participation. For example, McDonald *et al*. [43] used combined aerobic and resistance exercise to study post-stroke recovery of gait.

Improving functional recovery after stroke has largely centred on brain plasticity, with the role of skeletal muscle less studied [26]. Although we hypothesized that the modified rehabilitation program would attenuate muscle atrophy that would be evident in the stroke-targeted forelimb of the standard care group, we found no difference in either EDC or TBL muscle size between stroke-targeted and non-targeted forelimbs in either experimental group at 2 weeks after the conclusion of ER. The EDC, but not TBL, muscle was bilaterally larger after the modified enriched rehabilitation, as compared to standard care, although this did not influence recovery of reaching or spontaneous use of the stroke-targeted forelimb. We postulate that the bilateral change in EDC muscle size represents hypertrophy resulting from the voluntary exercise component of ER. Without sham control groups, some degree of bilateral EDC muscle atrophy in the SC group cannot be ruled out, although this seems less likely since, at the time of measurement, rats had been utilizing the limbs for support for 5 weeks since stroke onset. Given the observed changes in skeletal muscle, the rehabilitation protocol does appear to have value for studying the mechanistic contribution of muscle plasticity to post-stroke recovery and rehabilitation. In future, assessments at earlier timepoints after stroke would reveal if transient muscle atrophy develops with this model of stroke and is influenced by rehabilitation.

While we had hypothesized that skeletal muscle alterations would be accompanied by decreased weight gain in the ER group, given the increased opportunities for exercise, we found no change. Unfortunately, body weight is often not reported in environmental enrichment studies. Data from male, Sprague-Dawley rats for comparison yield mixed conclusions, ranging from a decrease to no change in rate of weight gain under enrichment conditions [63–66]. Many influential factors such as age, length and extent of enrichment, conditions used for the control housing comparison, and stroke severity could account for variable effects on weight gain. Differences in inherent drive to increase energy intake to match increased energy expenditure associated with activity as well as cage dynamics affecting food intake no doubt also contribute to the inconsistency. Some evidence also exists for strain and sex differences in effects of environmental enrichment on body weight [63, 66, 67].

Neither stroke nor the rehabilitation exerted major effects on metabolic fiber type in the EDC muscle at the timepoint studied. However, one notable alteration was that hybrid fibers, specifically Type IIb/x, occupied a greater proportion of the muscle cross-sectional area in the stroke-targeted forelimb despite no significant alteration in the number or size of hybrid fibers (S2 Table). This may be due to subtle increases in both measurements that when combined, resulted in a significant increase in area occupied by Type IIb/x fibers. The increase in hybrid fibers suggests that the muscle fiber type composition is in transition [68] in the stroke-targeted forelimb. The statistical trend for an increased proportion of EDC muscle IIb/x fibers in the stroke-targeted forelimb provides an additional clue suggestive of post-stroke muscle plasticity. In future experiments, sampling muscle immediately after the rehabilitation period would elucidate whether any such muscle fiber type switch contributed to the association between amount of reaching practice and reaching performance in the Montoya staircase observed at that time.

In contrast to our results, McDonald *et al*. [43] reported plantaris muscle atrophy in the stroke-targeted hindlimb at 6 weeks after photothrombotic stroke, with atrophy of fast-twitch Type IIb fibers, hypertrophy of Type I, IIa, and IIx fibers, and no change in the proportion of fiber types. A more intensive exercise regimen than ours, which included weight resistance and forced treadmill at VO_2_ peak, mitigated the stroke-induced abnormalities in fiber size. Similar to our experimental design, this study did not include sham controls, and thus the difference in muscle size between limbs may be contributed to by muscle hypertrophy on the side not targeted by stroke due to more reliance on this limb. Nonetheless, there are several key differences from our study, including the muscle studied and a larger infarct that could drive more muscle atrophy. A variety of reasons likely underpin differences in individual muscle susceptibility to atrophy after stroke, including the metabolic fiber composition of the specific muscle and influences such as age [69], nutritional status [37], and the extent of disuse atrophy [70]. Thus, different strategies for preserving or restoring muscle after stroke may be required depending on the contributions of individual factors.

Our study had strengths and limitations. Adherence to several key recommendations of the translational working group of the Stroke Recovery and Rehabilitation Roundtable [13] enhanced the rigour and translational value of our data. Examples include the chronic nature of the impairments induced as well as inclusion of at least two functional outcome measures and selection of adult rats rather than the adolescents more commonly used. While all rodent stroke models and tests of motor function have advantages and disadvantages for mimicking the heterogeneity of human stroke, our choices were among those recommended by this international group [13].

With respect to limitations, future studies would benefit from studying aged males and post-menopausal females [13]. While a key aim was to improve the translational value of the ER rehabilitation paradigm for application to future preclinical studies investigating the clinically relevant condition of malnutrition, this was unsuccessful since the modified ER failed to improve recovery of either skilled reaching or spontaneous use of the targeted forelimb after stroke. There is a need in future studies to more precisely quantify the amount of rehabilitation to ensure that the dosage of therapy is within a range that is clinically relevant. In addition, since the influence of ER on post-stroke muscle alterations was a secondary goal of the study, the sampling timepoint was not optimal to characterize the role of post-stroke muscle plasticity. Serial sampling of muscle at multiple times, such as acutely after stroke and directly following ER, as well as investigation of potential influence of sex, would be needed to fully examine effects on muscle size and metabolic fiber composition due to stroke and/or ER. The use of more intensive rehabilitation regimes and the study of several muscles of varying sensitivity to the effects of stroke would also yield a more powerful investigation.

In conclusion, the modified rehabilitation program did not promote recovery of skilled reaching or symmetry in spontaneous forelimb use when compared to a group exposed to stroke but no rehabilitation. While atrophy of the EDC muscle was not detected in the stroke-targeted forelimb in either experimental group at the post-stroke timepoint studied, the hybrid fibers did occupy a greater area in the whole muscle section. The rehabilitation bilaterally increased the EDC muscle size at 2 weeks after the cessation of rehabilitation, whereas the TBL muscle size was not influenced by either the stroke or the modified enriched rehabilitation. Based on the skeletal muscle changes observed, the rehabilitation program appears to have utility for future studies of the role of skeletal muscle plasticity in post-stroke recovery.

## Supporting information

S1 Table Physiological parameters measured during surgery. (PDF)

S2 Table Major hybrid fiber types in extensor digitorum communis muscle after stroke and enriched rehabilitation. (PDF)

S1 Dataset Original data. (XLSX)

S1 Fig Representative immunofluorescence images of myosin heavy chain (MHC) expression in extensor digitorum communis muscle. (A) Cocktail one containing BA-F8 identifying Type I fibers (blue), SC-71 identifying Type IIa fibers (green), and BF-F3 identifying Type IIb fibers (red); unstained fibers are labelled IIx. (B) An adjacent section stained with cocktail two containing SC-71 identifying Type IIa fibers (green) and 6HI identifying Type IIx fibers (red). Hybrid fibers are identified as staining positively for more than one fiber type and usually appear duller in staining.
(PDF)
