# Supplementary material for: A modified rehabilitation paradigm bilaterally increased rat extensor digitorum communis muscle size but did not improve forelimb function after stroke
Source: PLoS One. 2024 Apr 11;19(4):e0302008. doi: 10.1371/journal.pone.0302008 (PMC11008896; doi:10.1371/journal.pone.0302008)
Supplement: S1 Table — (PDF) [file pone.0302008.s001.pdf]

S1 Table. Physiological parameters measured during surgery.

Experimental group	SC (n=15)	ER (n=16)	P value
Oxygen Saturation (%)	98.3 ± 1.0	98.4 ± 1.2	0.645
Pulse (bpm)	333.4 ± 23.7	331.4 ± 21.6	0.806
Core Temperature (°C)	37.1 ± 0.3	37.1 ± 0.3	0.705

Data are shown as mean ± SD. ER, Enriched Rehabilitation; SC, Standard Care.
