# Supplementary material for: A modified rehabilitation paradigm bilaterally increased rat extensor digitorum communis muscle size but did not improve forelimb function after stroke
Source: PLoS One. 2024 Apr 11;19(4):e0302008. doi: 10.1371/journal.pone.0302008 (PMC11008896; doi:10.1371/journal.pone.0302008)
Supplement: S2 Table — (PDF) [file pone.0302008.s002.pdf]

S2 Table. Major hybrid fiber types in extensor digitorum communis muscle after stroke and enriched rehabilitation.

	Forelimb			
	SC Targeted (n=6)	SC Non-targeted (n=6)	ER Targeted (n=6)	ER Non-targeted (n=6)
Count (%)[1]				
Type IIa/x	3.80±1.02	4.74±3.67	4.09±0.72	5.00±2.09
Type IIb/x	7.36±1.39	6.70±2.18	5.82±1.15	4.45±2.09
Total counts[2]				
Type IIa/x	70±35	64±42	79±30	89±76
Type IIb/x	134±50	110±80	110±37	80±68
Fiber size (μm^2)[3]				
Type IIa/x	2847±744	2653±756	2985±962	2958±751
Type IIb/x	4570±678	4233±1140	4903±2061	4649±1301
Total area of fibers (mm^2)[4]				
Type IIa/x	0.19±0.08	0.16±0.09	0.23±0.09	0.23±0.15
Type IIb/x	0.59±0.19*	0.42±0.24	0.52±0.19*	0.36±0.26

Data are shown as mean ± SD (n= 6/group). ER. Enriched Rehabilitation; SC. Standard Care. ***** Signifies the larger area occupied by this hybrid fiber type in the stroke-targeted forelimbs of the pooled treatment groups ($P_{interaction}$ = 0.962, $P_{forelimb}$ = 0.039, $P_{treatment}$ = 0.562).

[1]The relative abundance of fiber types in the sampled regions.

[2] The number of fiber types in the whole muscle (calculated from [1] and the muscle area) adjusted for tendon area.

[3]Area of each fiber type measured in the sampled regions.

[4]Total area occupied by each fiber type (calculated from [3] and the muscle area) adjusted for tendon area.
