# Supplementary material for: A modified rehabilitation paradigm bilaterally increased rat extensor digitorum communis muscle size but did not improve forelimb function after stroke
Source: PLoS One. 2024 Apr 11;19(4):e0302008. doi: 10.1371/journal.pone.0302008 (PMC11008896; doi:10.1371/journal.pone.0302008)
Supplement: S1 Fig — (A) Cocktail one containing BA-F8 identifying Type I fibers (blue), SC-71 identifying Type IIa fibers (green), and BF-F3 identifying Type IIb fibers (red); unstained fibers are labelled IIx. (B) An adjacent section stained with cocktail two containing SC-71 identifying Type IIa fibers (green) and 6HI identifying Type IIx fibers (red). Hybrid fibers are identified as staining positively for more than one fiber type and usually appear duller in staining. (PDF) [file pone.0302008.s004.pdf]

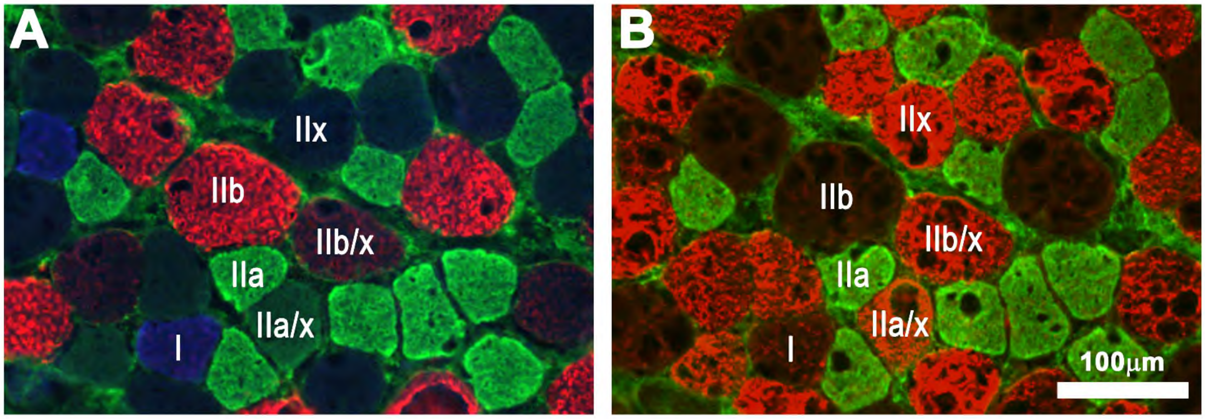

S1 Fig. Representative immunofluorescence images of myosin heavy chain (MHC) expression in extensor digitorum communis muscle. (A) Cocktail one containing BA-F8 identifying Type I Fibers (blue), SC-71 identifying Type IIa fibers (green), and BF-F3 identifying Type IIb fibers (red); unstained fibers are labelled IIx. (B) An adjacent section stained with cocktail two containing SC-71 identifying Type IIa fibers (green) and 6HI identifying Type IIx fibers (red). Hybrid fibers are identified as staining positively for more that one fiber type and usually appear duller in staining.
